# Dr. Wm. L. Rodman

**Published:** 1916-04

**Authors:** 


**Dr. Wm. L. Rodman,** Jefferson, 1879, Prof. of Surgery in the Medico-Chirurgical College of Medicine of Philadelphia, and a well known surgeon and author, died of pneumonia March 8, aged 58.

